# *Lepidium sativum* Secondary Metabolites (Essential Oils): In Vitro and In Silico Studies on Human Hepatocellular Carcinoma Cell Lines

**DOI:** 10.3390/plants10091863

**Published:** 2021-09-09

**Authors:** Shaimaa Nazir, Ahmed A. El-Sherif, Nour T. Abdel-Ghani, Mahmoud A. A. Ibrahim, Mohamed-Elamir F. Hegazy, Mohamed A. M. Atia

**Affiliations:** 1 Chemistry Department, Faculty of Science, Cairo University, Giza 12613, Egypt; shaimaa.nazeer@yahoo.com (S.N.); noureta2002@yahoo.com (N.T.A.-G.); 2 Computational Chemistry Laboratory, Chemistry Department, Faculty of Science, Minia University, Minia 61519, Egypt; m.ibrahim@compchem.net; 3 Chemistry of Medicinal Plants Department, National Research Centre, Giza 12622, Egypt; 4 Molecular Genetic and Genome Mapping Laboratory, Genome Mapping Department, Agriculture Genetic Engineering Research Institute (AGERI), Agriculture Research Centre (ARC), Giza 12619, Egypt

**Keywords:** garden cress, *Lepidium sativum*, hepatocellular carcinoma, GC–MS analysis, apoptosis, qPCR, molecular docking

## Abstract

Hepatocellular carcinoma (HCC) is the most common primary liver cancer and the greatest cause of cancer-related death in the world. Garden cress (*Lepidium sativum*) seeds have been proven to possess extraordinary antioxidant, anti-inflammatory, hypothermic, and analgesic properties. In this study, in vitro cytotoxic efficiency evaluation of *L. sativum* fractions was performed against two hepatocellular carcinoma cell lines (HuH-7 and HEPG-2), and the expression of some apoptotic genes was explored. In addition, the chemical composition of a potent extract of *L. sativum* was analyzed using gas chromatography coupled with mass spectrometry. Then, molecular docking analysis was implemented to identify the potential targets of the *L. sativum* components’ most potent extract. Overall, the *n*-hexane extract was the most potent against the two HCC cell lines. Moreover, these cytotoxicity levels were supported by the significant downregulation of EGFR and BCL2 gene expression levels and the upregulation of SMAD3, BAX, and P53 expression levels in both HuH-7 and HEPG2 cell lines. Regarding *L. sativum*’s chemical composition, GC–MS analysis of the *n*-hexane extract led to the identification of thirty compounds, including, mainly, hydrocarbons and terpenoids, as well as other volatile compounds. Furthermore, the binding affinities and interactions of the *n*-hexane fraction’s major metabolites were predicted against EGFR and BCL2 molecular targets using the molecular docking technique. These findings reveal the potential use of *L. Sativum* in the management of HCC.

## 1. Introduction

Liver cancer is the second highest cause of cancer-related mortality worldwide. Meanwhile, primary liver cancer comprises intrahepatic cholangiocarcinoma (iCCA) and hepatocellular carcinoma (HCC) forms, with HCC being the most predominant [1]. HCC constitutes a global crisis, and its epidemiological data fluctuate from place to place. HCC is estimated to be the sixth most prevalent type of cancer worldwide; in Egypt, it represents the fourth most widespread cancer [2]. The prevalence of HCC is rising dramatically, especially in Africa and Asia. For example, in Egypt, health officials consider HCC the most critical health problem. Remarkably, the incidence of HCC has increased approximately two-fold over the last decade [3]. Chronic HBV (Hepatitis B Virus) infection, autoimmune hepatitis, chronic alcohol use, obesity, diabetes mellitus, and hepatitis C virus (HCV) sequelae are all risk factors for HCC in Egypt [4]. In actuality, extensive attempts have been made to resolve this problem through the development of novel treatments that can replace the current conventional therapies, such as chemotherapy, which is still the primary approach used to treat liver cancer [5].

Notwithstanding, chemotherapy frequently stimulates many side effects, including pain, hepatotoxicity, renal toxicity, and cardiotoxicity [6]. Alternatively, natural products are regarded as the most outstanding sources in drug discovery [7,8]. Humans have used natural products/herbal remedies to preserve health, prevent illnesses, and promote mental and physical well-being for decades [9]. Many studies have shown that some natural items coupled with chemotherapeutic medicines can have chemoprotective and/or synergistic effects on reducing cancer chemotherapy-related adverse effects and increasing therapeutic efficiency [10].

*Lepidium sativum* or curly cress is a member of the Cruciferae family (Brassicaceae), widely grown in India, Europe, and the United States. The seeds of *L. sativum* have been found to contain significant ratios of alkaloids, flavonoids, glycosides, polyketides, vitamins, minerals, proteins, fats, and carbohydrates, which explain its potency against different diseases [11,12]. *L. sativum* seeds consist of 24% oil; of this percentage, 32% is represented by a-linolenic acid and 12% by linolenic acid. Remarkably, the main constituents identified in the oil were 7,10-hexadecadienoic acid, 7,10,13-hexadecatrienoic acid, behenic acid, and 11-octadecenoic acid [13]. Moreover, this oil is highly stable due to its high content of phytosterols and antioxidants [14]. In traditional medicine, *L. sativum* is regarded as a therapy for many diseases, such as arthritis, diabetes mellitus, and hepatitis [15]. Furthermore, *L. sativum* is known to have antihypertensive, diuretic, anti-inflammatory, analgesic, anticoagulant, antirheumatic, hypoglycemic, laxative, prokinetic, antidiarrheal, and antispasmodic properties [11]. In addition, *L. sativum* oil has been found to exert inhibitory synergistic influences on thromboxane B2 and platelet aggregation levels, especially in the lung and spleen of Wistar rats [16]. Moreover, various extracts of *L. sativum* seeds have been proven to exert antimicrobial activity against many infectious bacteria and fungi [17]. In addition, recent reports have demonstrated that *L. sativum* extracts exert anticancer activity against various types of cancer cell lines, such as a bladder cancer cell line [18], K562 leukemia cell lines [19], and breast cancer cells (MCF-7) [20]. Meanwhile, *L. sativum* seed extracts have been reported to possess a significant protection ability against chloroform-induced liver damage [21]. To date, the hepatoprotective mechanism of *L. sativum* is still ambiguous, but it may be due to the capability to prevent liver lipid peroxidation [22]. Therefore, the current study aimed to characterize the chemical constituents and the cytotoxic activity of *L. sativum* against two hepatocellular carcinoma cell lines. Additionally, their mechanism of action was determined through characterization of the expression of some apoptotic gene(s). Finally, molecular docking tools were utilized to predict the binding modes and affinities of the major metabolites against potential biological targets.

## 2. Results

### 2.1. Extraction 

The aqueous methanol (80%) of a fine powder of *L. sativum* seeds (1 kg) was successively fractionated via liquid–liquid techniques to afford five different solvents, i.e., methylene chloride (43 g), *n*-hexane (33 g), ethyl acetate (15 g), butanol (13 g), and methanol (12 g), containing compounds with varying polarity.

### 2.2. Cell Cytotoxicity

**Figure 1:**
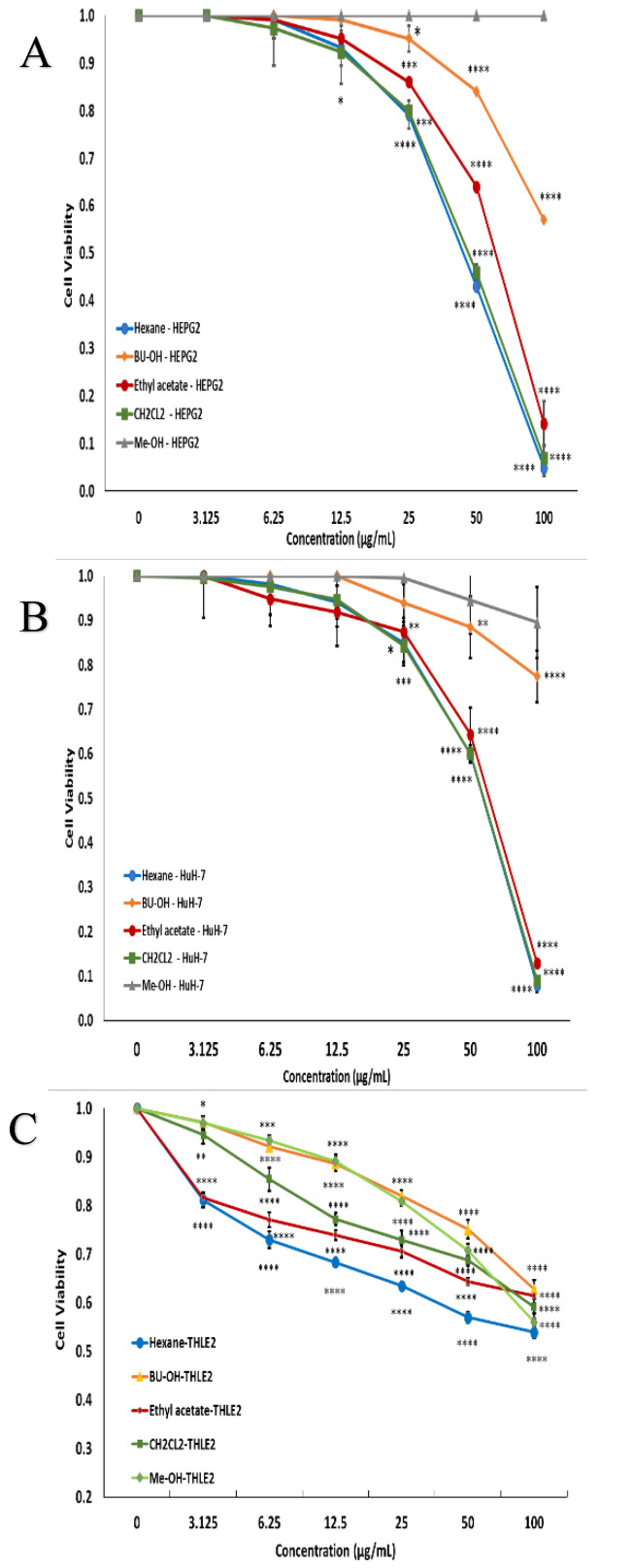
Effect of different concentrations of methylene chloride, butanol, methanol, ethyl acetate, and *n*-hexane extracts on the cellular proliferation of HEPG2 (**A**), HuH-7 (**B**), and THLE 2 (**C**) cell lines following 48 h of treatment. Values are expressed as the mean ± SD of 3 separate experiments performed in quadruplicate. Statistical analysis was carried out utilizing analysis of variance (ANOVA) followed by post-hoc test, and Dunnett t-test was used for comparison with the control group. All tests were two-tailed. A *p*-value < 0.05 was considered significant (* *p* ≤ 0.05, ** *p* ≤ 0.01, *** *p* ≤ 0.001, **** *p* ≤ 0.0001).

The effects of different concentrations of methylene chloride, butanol, methanol, ethyl acetate, and *n*-hexane extracts on the cellular proliferation of HEPG2 and HuH-7 cell lines following 48 h of treatment were studied. The cell cytotoxicity results revealed a concentration-dependent cytotoxic effect of *n*-hexane (IC_50_ = 45 µg/mL), methylene chloride (IC_50_ = 47 µg/mL), and ethyl acetate (IC_50_ = 63.8 µg/mL) on HEPG 2 cells (Figure 1A), while on HuH-7 cells, *n*-hexane (IC_50_ = 59.3 µg/mL), methylene chloride (IC_50_ = 59 µg/mL), and ethyl acetate (IC_50_ = 63.5 µg/mL) were compared to a non-treated control (Figure 1B) using the normal cell line, THLE 2 (Figure 1C). Thus, the *n*-hexane extract was observed to be the most effective.

### 2.3. Gas Chromatography (GC) Analysis

The GC–MS analysis of the *n*-hexane extract of *L. sativum* revealed the identification of thirty compounds that represented 97.67% of the total mass, including hydrocarbons (69.16%) in addition to terpenoids (18.80%) and other volatile compounds (9.71%). The hydrocarbons were characterized as abundant compounds in the *n*-hexane extract of this plant, with fifteen identified compounds. From \the characteristic hydrocarbons, linolenic acid, methyl ester (26.44%), oleic acid, methyl ester (11.76%), 11-eicosenoic acid, methyl ester (4.80%), and 6,9,12,15-docosatetraenoic acid, methyl ester (4.15%) were identified as the major compounds, while 2-[(9Z,12Z)-9,12-Octadecadien-1-yloxy]ethanol (0.52%) was identified as a minor one.

Meanwhile, terpenoids, with a concentration of (18.80%), were considered the second most identified class of compounds, including mono and sesqui-terpenoids, with the complete absence of diterpenoids. Five monoterpene compounds were identified from among the characterized terpenoids and represented (13.38%) of the overall mass. Methyleugenol (4.77%) and anisole, p-propenyl-, cis- (4.13%) were identified as the main monoterpenes, while ç-asarone (0.68%) was identified as a minor compound. Furthermore, sesquiterpenes were observed at remarkable concentrations (5.42%), involving only four compounds. Caryophyllene was identified as a major compound, with a concentration of 3.82% among the identified sesquiterpenes, but sesquisabinene isomer was identified as a minor one (0.74%). In addition to hydrocarbons and terpenes, other volatile compounds were identified at minor concentrations (9.71%). These identified compounds include three aromatic compounds, namely 2-methoxy-4-[(1e)-1-propenyl]phenol (1.15%), 9,12,15-octadecatrienoic acid, 2-phenyl-1,3-dioxan-5-yl ester (1.00%), and propanoic acid, 2-(3-acetoxy-4,4,14-trimethylandrost-8-en-17-yl)- (1.19%), and one tocopherol, ç-tocopherol (3.55%). Additionally, two nitrogenated compounds, 1H-purin-6-amine, [(2-fluorophenyl) methyl]- (1.79%) and 10-nethoxy-Nb-alpha-methylcorynantheol (1.03%) were identified (Table 1).

### 2.4. Effect of L. sativum Extracts on Expression of EGFR and BCL2, TGF-β, SMAD3, BAX, and P53 Genes 

**Figure 2:**
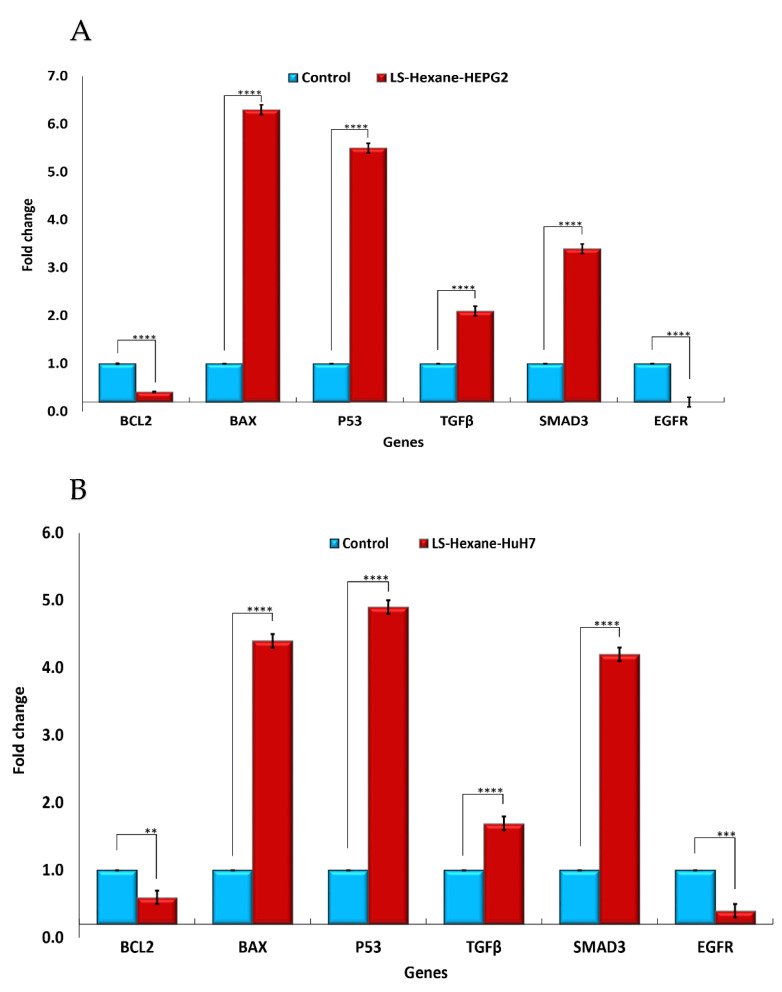
Gene expression levels after treating hepatocellular carcinoma cells with *L. sativum n*-hexane extract. (**A**): Expression of BCL2, BAX, P53, TGF-β, SMAD3, and EGFR in HEPG2 cells. Blue: untreated control, red: hexane extract. (**B**): Expression of BCL2, BAX, P53, TGF-β, SMAD3, and EGFR in HuH-7 cells. Blue: untreated control, red: hexane extract. Statistical analysis was performed using analysis of variance (ANOVA) followed by post-hoc test, and Dunnett t-test was used for comparison with the control group. All tests were two-tailed. A *p*-value < 0.05 was considered significant (** *p* ≤ 0.01, *** *p* ≤ 0.001, **** *p* ≤ 0.0001).

To investigate the effects of *L. sativum n*-hexane extract on HCC inhibition or development, we presumed that *L. sativum* extract might re-modulate the expression levels of some apoptotic gene markers. Apoptosis is a biological pathway controlled by various signal transduction actions, including the regulation of the P53, BAX, and BCL2 genes [23]. Generally, the expression patterns obtained during this study using qPCR-based profiling of the proapoptotic gene markers (P53 and BAX) showed significant increases in the mRNA transcripts of these genes in both the HuH-7 and HEPG2 cell lines compared to the untreated control (Figure 2A,B). Regarding the P53 gene expression level, the treatment with *n*-hexane extract in the HEPG2 cell line showed increased expression, with a fold change of 5.5 ± 0.1 and upregulation in the HuH-7 cell line by 4.9±0.1 fold. Similarly, regarding the BAX gene expression level, the treatment with *n*-hexane extract in the HEPG2 cell line led to an increase in expression, with a fold change of 6.3±0.1, and upregulation in the HuH-7 cell line by 4.4 ± 0.1 fold.

In contrast, the BCL2 gene is considered the most vital antiapoptotic protein, and many reports have confirmed its overexpression level in several human tumors [24]. For the *L. sativum n*-hexane extract, BCL2 showed a consistent downregulation pattern in the HEPG2 cell line, with a fold change of 0.41 ± 0.01, and downregulation in the HuH-7 cell line by 0.6 ± 0.1 fold (Figure 2 A and B). 

Moreover, the TGF-β signal transduction pathway is considered an important regulator of hepatocellular carcinoma and also accelerates liver fibrosis [25]. Under the TGF-β signal transduction pathway, the SMAD2 and SMAD3 genes were found to be critical intracellular mediators, even reflecting tumor-inhibitory or tumor-inducing effects [25]. The current results revealed that the expression level of TGF-β was upregulated upon treatment with the *n*-hexane extract of *L. sativum* in the HEPG2 cell line, with a fold change of 2.1 ± 0.1, and it was upregulated in the HuH-7 cell line by 1.7 ± 0.1 fold. Meanwhile, SMAD3 gene expression was significantly upregulated after treatment with *n*-hexane extract in the HEPG2 cell line, with a fold change of 3.4±0.1, and it was also raised in the HuH-7 cell line by 4.2 ± 0.1 fold.

The EGFR pathway is essential in liver regeneration, cirrhosis, and hepatocellular carcinoma. Its overexpression was confirmed to play a pivotal tumor-promoting role in HCC development [26]. The current study found a significant downregulation in EGFR gene expression upon exposure to the n-hexane extract in the HEPG2 cell line, with a fold change of 0.2 ± 0.1, and downregulation in the HuH-7 cell line by 0.4±0.1 folds.

### 2.5. Molecular Docking Calculations and Pathway Enrichment Analysis (PEA)

**Figure 3:**
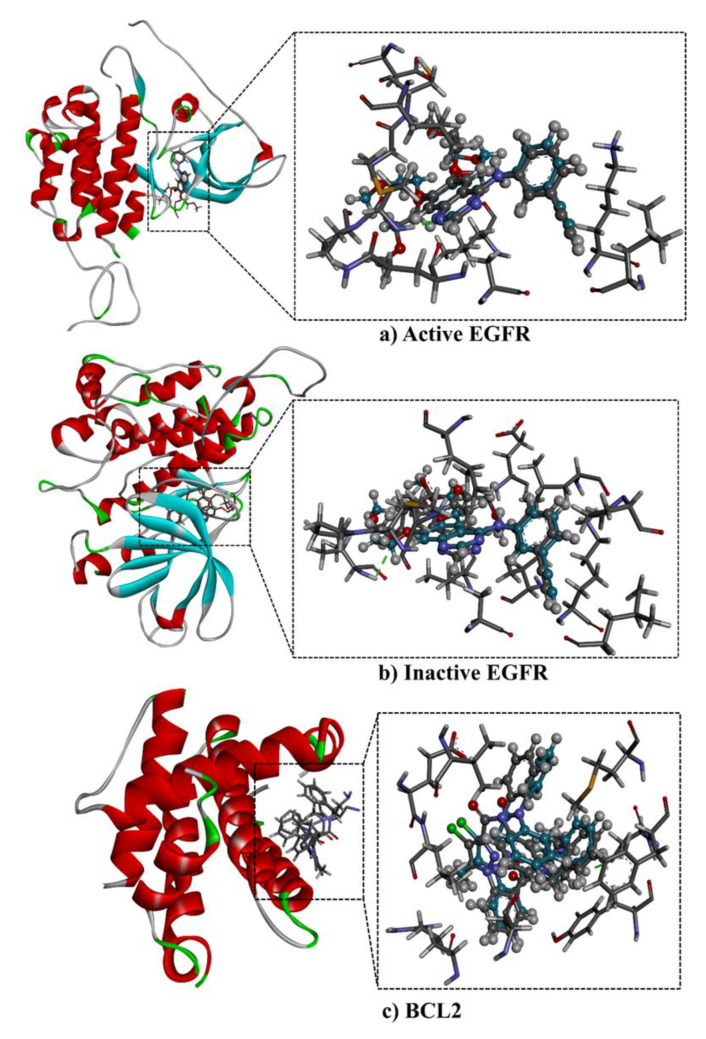
3D representations of the predicted (in grey) and crystal (in cyan) binding modes of (**a**) erlotinib (AQ4) with the active conformation of EGFR (PDB code: 1M17), (**b**) erlotinib with the inactive conformation of EGFR (PDB code: 4HJO), and (**c**) DRO with BCL2 (PDB code: 2W3L).

The binding mechanisms and affinities of the main metabolites in the *n*-hexane extract with EGFR and BCL2 were predicted using molecular docking. Prior to in silico prediction, the performance of the AutoDock4.2.6 software with the employed parameters to predict the binding modes of EGFR and BCL2 inhibitors was assessed based on the available experimental data. The co-crystalized EGFR and BCL2 ligands—namely AQ4 and DRO, respectively—were re-docked, and the predicted binding modes were compared to the corresponding experimental poses (Figure 3). Comparing the predicted and experimental binding modes revealed that the AutoDock4.2.6 software accurately reproduced the crystal structure and the observed binding modes of AQ4 and DRO (Figure 3). The excellent performance of AutoDock4.2.6 in predicting the crystal binding mode of erlotinib (AQ4) with EGFR is in line with the results of our previous study [27].

**Figure 4:**
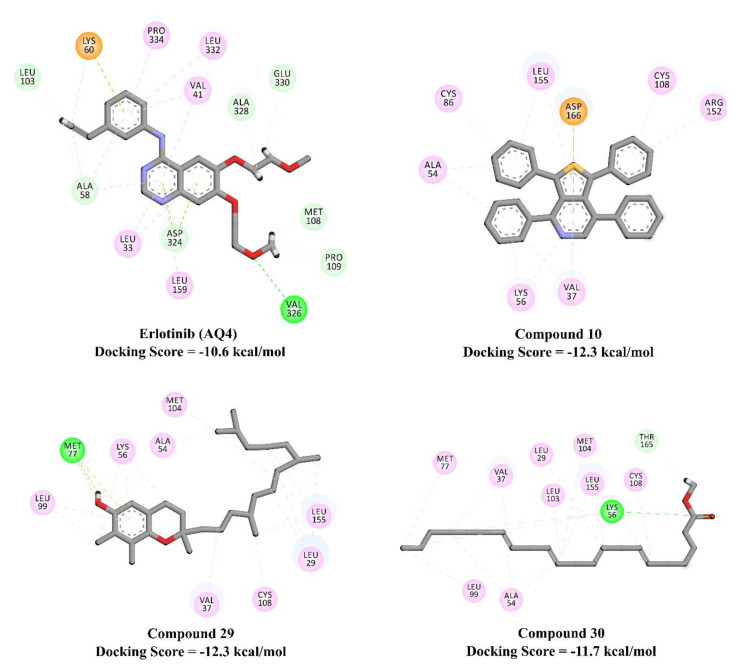
2D representations of the binding modes of potent *n*-hexane extract major metabolites inside the active site of inactive EGFR (PDB code: 4HJO). Interactions: conventional hydrogen bond (green), carbon–hydrogen bond (pale green), pi–sigma and pi–pi (violet), pi–sulfur (yellow), alkyl and pi–alkyl (pale violet).

Using the AutoDock4.2.6 software, *n*-hexane extract major metabolites were docked against EGFR and BCL2, and their binding affinities and modes were predicted. Predicted docking scores of the investigated metabolites are listed in Table 2, and 2D representations of their binding modes inside the active sites of the EGFR and BCL2 proteins are depicted in Figure 4.

Compared to the binding affinity of erlotinib (AQ4) with inactive EGFR (−10.6 kcal/mol), compounds 10, 29, and 30 demonstrated higher docking scores, with values of −12.3, −12.3, and −11.7 kcal/mole, respectively. In contrast, the rest of the compounds exhibited relatively weak binding affinities, with values in the range of −5.2 to −10.3 kcal/mol. Inspecting the predicted binding modes revealed that the high potency of inactive EGFR inhibitors can be attributed to their ability to participate in pi-based and alkyl interactions, including pi–alkyl, pi–anion, and pi–sulfur interactions (Figure 4).

**Figure 5:**
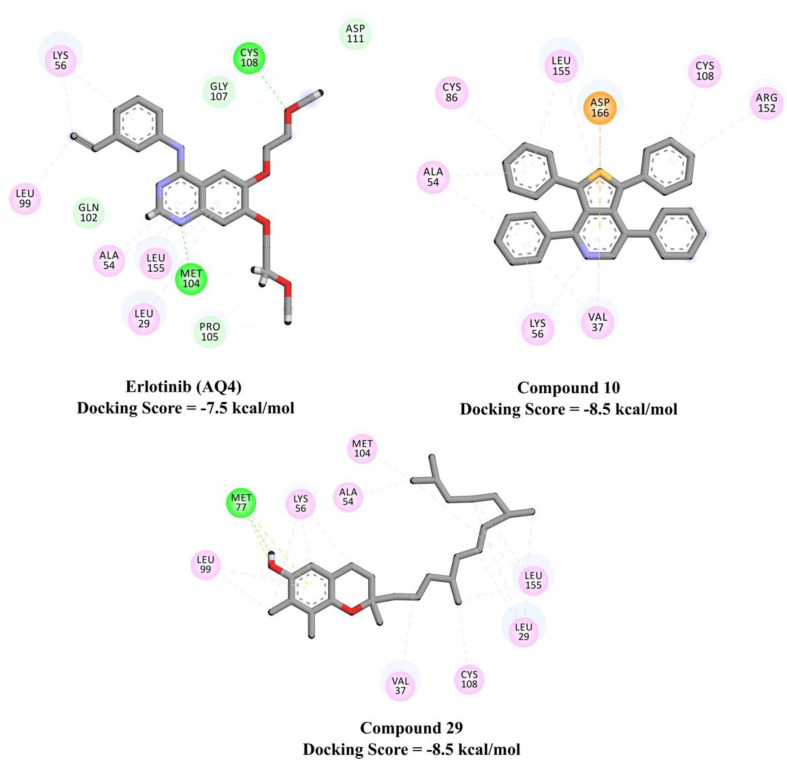
2D representations of the binding modes of potent *n*-hexane extract major metabolites inside the active site of inactive EGFR (PDB code: 4HJO). Interactions: conventional hydrogen bond (green), carbon–hydrogen bond (pale green), pi–sigma and pi–pi (violet), pi–sulfur (yellow), alkyl and pi–alkyl (pale violet).

The investigated metabolites showed relatively weaker docking scores towards active EGFR (PDB code: 1M17), with values in the range of −2.4 to −8.5 kcal/mol, compared to the inactive EGFR. The same trend of a lower binding affinity towards active EGFR was also observed for erlotinib (AQ4), with docking scores of −7.5 and −10.6 with active and inactive conformations of EGFR, respectively. Interestingly, among the investigated metabolites, compounds 10 and 29 exhibited higher binding affinities with active EGFR than erlotinib (docking scores of −8.5, −8.5, and −7.5 kcal/mol for compounds 10, 29, and erlotinib, respectively). The pi-based interactions and alkyl interactions dominated the binding affinity of compounds 10 and 29 with active EGFR (Figure 5).

Considering the predicted binding affinity of the co-crystalized ligand DRO with BCL2 (docking score of −9.9 kcal/mol), the major metabolites from the *n*-hexane extract showed much lower docking scores, with values in the range of −3.4 to −7.7 kcal/mol.

For pathway enrichment analysis (PEA) and Reactome mining, the results were in line with the results obtained regarding the expression levels of some apoptotic and signal transduction gene markers. Interestingly, Reactome representation based on Boolean network modeling confirmed that the EGFR pathway was among the most enriched pathways by compounds 10 and 29; this pathway is vital in liver regeneration and hepatocellular carcinoma development. Notably, the programmed cell death pathway was not stimulated/influenced by compounds 10 and 29, revealing that these compounds had no or a negligible direct effect on inducing programmed cell death in cancerous cells (Figures S1 and S2).

## 3. Discussion

In Egypt, liver cancer is one of the most prevalent types of cancer among both men and women, according to the National Cancer Registry Program (NCRP) of the National Cancer Institute (NCRP, 2018) [4]. In addition, hepatitis C is a major public health problem, with the highest global prevalence of hepatitis C virus (HCV); the most common genotype in Egypt is genotype 4 [28].

Chemotherapy-associated side effects due to its non-selectivity and the resistance of cancer cells remain a major obstacle in cancer treatment. Natural therapies, such as plant-derived compounds, are a promising addition to cancer therapy. Solutions to controlling the initiation and progression of cancer are of great value [29]. The current research aimed to evaluate the potential use of garden cress in inhibiting liver cancer at the in vitro level and to explore its possible mechanism of action.

Previous research proved that *L. sativum* extracts display a cytotoxic effect against different types of cancer, including leukemia [19] and breast cancer [20]. In agreement with this, the current study showed a concentration-dependent cytotoxic effect of methylene chloride (47 ug/mL), ethyl acetate (63.8 ug/mL), and *n*-hexane (45 ug/mL) extracts on HEPG2 cells, while on HuH-7 cells, methylene chloride (59 ug/mL), ethyl acetate (63.5 ug/mL), and *n*-hexane (59.3 ug/mL) from *L. sativum* extracts were compared to a non-treated control.

Additionally, the obtained results revealed a harmony with previous different research studies, in which the cytotoxic effects of a methanolic extract of *L. sativum* seeds on the bladder cell line (ECV-304) have been reported by Al-Fatimi et al. (2005) [18]. Moreover, Asalani and colleagues (2015) found that the aerial parts of the *L. sativum* plant exert anticancer activity against the K562 leukemia cell line [19]. Furthermore, the aqueous extract of *L. sativum* seeds induced apoptosis and necrosis in breast cancer cells (MCF-7), causing a significant time- and dose-dependent reduction in cell viability [20]. Garden cress leaf extract showed a strong antiproliferative effect against CAL-27 cells, mediated through the apoptosis process [30]. 

Recently, Abd El-Kaream (2019) reported that garden cress extracts exhibited excellent antioxidant, chemopreventive, and chemotherapeutic effects against DMBA-induced hepatotoxicity; furthermore, the researcher found that *L. sativum* juice and powder presented a hepatoprotective effect against carbon tetrachloride and 2-amino-3-methylimidazole-4, 5-quinoline [31]. Furthermore, Abuelgasim et al. (2008) found that *L. sativum* juice prevents hepatocarcinogenesis by increasing the UDP-glucuronyl-transferase-2 and detoxifying carcinogens. Additionally, it prevents liver injury by inhibiting aspartate aminotransferase, alanine aminotransferase, nitric oxide, leukotriene B4, interleukin 2, tumor necrosis factor, and transforming growth factor β [32]. Meanwhile, Raish et al. (2016) noted that the ethanolic extract of *L. sativum* exhibited a hepatoprotective effect by reducing aspartate aminotransferase, alkaline phosphatase, alanine aminotransferase, gamma glutamyl transferase, and bilirubin, inhibiting NF-ᴋβ activity, reducing myeloperoxidase content, decreasing thiobarbituric acid reactive substance, downregulating interleukin-6, tumor necrosis factor, caspase-3, iNOS, and HO-1, and upregulating interleukin-10 and BCL2 expression [21].

*L. sativum n*-hexane extract contains tocopherol, linoleic acid, oleic acid, linolenic acid, methyleugenol, and anisole, according to GC–MS analysis. Garden cress is high in antioxidants such as vitamins A, B, C, E, isotiosinat, omega-3 fatty acids such as alpha-linolenic acid, and glucosinolates, as well as glucosinolates, which might reinforce its anticancer effects due to their antioxidant activity [19]. From another point of view, the anticancer effect might be attributed to the isothiocyanates, specifically benzyl isothiocyanate. Apoptosis was induced in MCF-7 upon treatment with 25% and 50% extract, while necrosis occurred after exposure to elevated extract concentrations (75%) [20]. In agreement with previous research, the present result indicates the presence of different antioxidant compounds in *L. sativum* extracts, which contribute to its antitumor activity.

On the other hand, the EGF receptor pathway plays a crucial role in different cancer types. In particular, the EGFR pathway is critical in liver regeneration, cirrhosis, and hepatocellular carcinoma (HCC) [33]. In HCC, EGFR is overexpressed in liver macrophages, where it has a tumor-promoting role [34]. Komposch and Sibilia (2015) found that the EGFR pathway in liver cells prevents the overproduction of interleukin 1 beta (IL-1β) and compensatory proliferation, suggesting its antitumorigenic role [35]. EGFR upregulation occurs in 68% of HCC cases, correlating with tumor aggressiveness, metastasis, and poor prognosis. Lanaya et al. (2014) discovered that deleting EGFR in liver cells increased the frequency of HCC, but deleting it in Kupffer cells and/or liver macrophages dramatically decreased the development of HCC in mice. They also discovered that animals without EGFR in macrophages had a reduced risk of hepatocarcinogenesis, but mice lacking EGFR in hepatocytes had a higher risk of HCC due to greater liver cell damage. EGFR is necessary for liver macrophages to produce interleukin-6 in response to interleukin-1 stimulation, which causes liver cell proliferation and HCC. The loss of EGFR in hepatocytes causes HCC, and EGFR-positive liver macrophages are linked to poor overall survival in HCC patients [34]. Since EGFR is a primary driver of tumorigenesis and is recognized as a resistance biomarker, and EGFR signaling is involved in the regulation of different metabolic pathways that are crucial for cancer cell proliferation [36], the current results regarding its downregulation by Lepidium sativum extracts might be one of the mechanisms involved in its cytotoxic activity. 

Remarkably, the TGF-β signal transduction pathway is regarded as a central regulator of liver cancer [25] due to its tumor-suppressive properties and/or its pro-metastatic effects [37]. In clinical practice, increased TGF-β in the early stages is associated with a promising prognosis, but in progressed tumors, it is associated with increased tumor invasiveness and dedifferentiation, suggesting that TGF-β initially restrains hepatocellular carcinoma via its tumor-suppressive effects but may later aggravate the malignancy due to its pro-oncogenic functions [38]. Therefore, in contrast to EGFR, our obtained results revealed the significant upregulation of the TGFβ and SMAD3 genes in both the HUH7 and HEPG2 cell lines compared to the untreated control. These results might explain the dual role of TGF-β, acting either as a suppressor during early stages or contributing to tumor progression during late stages, when cells evade its cytostatic effects. In other words, TGF-β’s protumorigenic effects include epithelial–mesenchymal transition (EMT) and alterations in the plasticity of tumor cells [39,40].

Furthermore, TGF-β is a pleiotropic cytokine that communicates with intracellular SMAD proteins and membrane receptors. Therefore, in early tumorigenesis, TGF-β has a tumor suppressor effect. On the other hand, it has a protumorigenic role in the late stages, promoting invasiveness and metastasis [41,42]. Massagué (1998) found that the expression of the central TGF-β signaling transducer, SMAD3, reduced the susceptibility to HCC in a chemically induced model murine. SMAD3’s protective effect involves apoptosis induction by repressing BCL2 transcription. The proapoptotic effect of SMAD3 involves TGF-β signaling and activation, which selectively take place in liver cancer cells. Thus, SMAD3 aids the tumor suppression effect of TGF-β by serving as a mediator of TGF-β-induced apoptosis [43].

Moreover, SMAD3 has excellent potential as a gene therapeutic agent for liver cancer treatment. TGF-β/SMAD3 signaling plays a direct role in controlling the cellular level of BCL2, which is crucial for TGF-β-mediated apoptosis in the liver. SMAD3 downregulates the expression of BCL2, inducing apoptosis through TGF-β [44]. In agreement with this, the current work explains the observed cytotoxic effect of *L. sativum* extracts via the upregulation observed in the TGF-β and SMAD3 genes. 

On the other hand, P53 is a core gene that regulates the cell cycle and suppresses cancer progression [45]. It has been reported that the P53 tumor suppressor gene exhibits an upregulation pattern, which promotes the cell apoptosis process through interaction with the BCL2 gene product [46]. The BCL2/BAX ratio proves the ability of *L. sativum* in apoptosis induction. In agreement with previous reports, our results demonstrated that the hexane extract of *L. sativum* significantly downregulated the mRNA expression patterns of the EGFR and BCL2 genes, which explains its proliferation effect. On the other hand, *L. sativum* extract significantly upregulated TGFβ, SMAD3, BAX, and P53 expression levels in both the HuH-7 and HEPG2 cell lines compared to the untreated control. Consequently, *L. sativum* inhibited the proliferation and growth of HCC cell lines and increased the apoptosis of these cells.

Molecular docking calculations revealed higher binding affinities of compounds 10 and 29 against active EGFR compared to erlotinib (docking scores of −8.5, −8.5, and −7.5 kcal/mol, respectively). 

## 4. Materials and Methods

### 4.1. Method of Extraction

Seeds of *L. sativum* were purchased from a local herbal supplier (Ragab El-Attar Herbal Store, Cairo, Egypt). A copy of the purchased seeds used in the current study was deposited in the Botany Department’s herbarium at the Faculty of Science, Cairo University, under voucher specimen number (10001). The *L. sativum* species were taxonomically authenticated by Prof. Dr. Reem Samir (Professor of Botany, Faculty of Science, Cairo University, Cairo, Egypt). The seeds were manually screened, and then uniformed ones were ground using an electrical grinder. At room temperature, the fine powder (1 kg) of *L. sativum* seeds was extracted with aqueous methanol (80%). Next, the seeds of *L. sativum* were extracted, and extracts were evaporated in vacuo at 45 °C, followed by the successive liquid–liquid extraction of the crude extract with *n*-hexane (33 g), CH_2_Cl_2_ (43 g), EtOAc (15 g), and BuOH (13 g).

### 4.2. Cell Line Culture

HEPG2 and HuH-7 hepatocellular carcinoma cells were obtained from the American Type Culture Collection (ATCC), USA. The cells were maintained by serial subculturing into 75 cm^3^ flasks in Dulbecco’s modified eagle medium (DMEM; Invitrogen, Carisbad, CA, USA) supplemented with 10% heat-inactivated fetal bovine serum (FBS), 1mM sodium pyruvate, 2 mM L-glutamine (Invitrogen), and 1% penicillin G/streptomycin (Invitrogen). In a water-jacketed incubator, the cells were maintained in monolayer culture at 37 °C under a humidified atmosphere of 5% CO_2_ (Revco, RCO 3000 TVBB, USA). The cell lines were repeatedly subcultured in order to keep them in the exponential growth phase. Sterile conditions were achieved under an equipped laminar flow (Microflow Laminar Flow Cabinet, Hampshire SP105AA, U.K.).

### 4.3. MTT Assay

The vitality of the cells was evaluated by performing the MTT (3-(4,5-dimethylthiazol-2-yl)-2,5-diphenyltetrazolium) bromide assay according to our protocol [47]. Media were aspirated, and fresh medium (without serum) was added to the cells with various concentrations (0, 3.125, 6.25, 12.5, 25, 50, and 100 µg/mL) of plant extracts and incubated for 48 hours. Cell-free controls (as a blank) were used in each plate that contained MTT plus compounds in culture media to correct any reduction of MTT in the absence of cellular mitochondria [48]. 

### 4.4. Gas Chromatography

Analysis of *L. sativum n*-hexane extract’s chemical composition was achieved using a mass spectrometer (Trace GC-TSQ Thermo Scientific, Austin, TX, USA) connected to a direct capillary column, the TG-5MS (30 m × 0.25 mm × 0.25 μm film thickness). The temperature of the column was initially 50 °C, followed by an increase by 5 °C / min to 250 °C, held for 2 min; followed by an increase to reach 300 °C by 30 °C /min, held for 2 min. The instrument conditions, sample preparation, and sample injection procedures were derived from Abd El-Kareem et al., 2016 [49]. The components’ retention time (RT) and mass spectra (MS) were compared to those in the WILEY 09 and NIST 14 mass spectral databases [49]. 

### 4.5. Quantitative Real-Time Polymerase Chain Reaction (qRT-PCR) 

Total RNA was extracted from HuH-7 and HEPG2 cells using the RNeasy Mini Kit procedure (Qiagen, Valencia, CA, USA). The High-Capacity cDNA Reverse Transcription Kit (Applied Biosystems, CA, USA) was used for reverse transcription, and the GoTaq® qPCR Master Mix (Promega, Madison, Wisconsin, USA) was used for real-time PCR to detect the expression of TGF, SMAD3, EGFR, BAX, BCL2, and P53 genes on a 7900HT Fast Real-Time PCR System (Applied Biosys-tems, USA). The amplification settings were as follows: one cycle at 95 °C for 10 min, followed by 40 cycles at 95 °C for 15 s and one min at 60 °C. Invitrogen provided all of the primers (Carlsbad, CA, USA). The primer sequences used were as follows: TGFβ (F: GGACAC CAACTATTGCTTCAG, R: TCCAGGCTCCAAATGTAGG), SMAD3 (F: GCCTGTGCTGGAACATCATC; R: GCCTGTGCTGGAACATCATC), EGFR (F: GCAATATCAGCCTTAGGTGCGGCTC; R: GCAATATCAGCCTTAGGTGCGGCTC), BAX (F: CTACAGGGTTTCATCCAG; R: CCAGTTCATCTCCAATTCG), BCL2 (F: GTGGATGACTGAGTACCT; R: CCAGGAGAAATCAAACAGAG), P53 (F: GTATTTCACCCTCAAGATCC; R: TGGGCATCCTTTAACTCTA), β-Actin (F: TTCCAGCCTTCCTTCCTGG; R: TTGCGCTCAGGAGGAGCAAT). The ^ΔΔ^Ct technique [50] was used to perform quantitative data analysis. The expression levels were adjusted to -Actin and presented as a percentage.

### 4.6. Molecular Docking and Pathway Enrichment Analysis (PEA)

Prediction of the binding affinities and modes of the major metabolites with EGFR and BCL2 was performed using AutoDock4.2.6 software [50]. Both active and inactive conformations of EGFR were considered in the current study. The resolved crystal structures of active EGFR, inactive EGFR, and BLC2 (PDB codes: 1M17 [51], 4HJO [52], and 2W3L [53], respectively) were downloaded and prepared for docking calculations. Protein preparation included: (i) cleaning of water molecules, ions, and ligands, and (ii) investigation of the protonation state and addition of missing hydrogen atoms using the H++ server [54]. The docking parameters were kept as the default, with an energy evaluation maximum number (*eval*) of 25,000,000 and a genetic algorithm (*GA*) run of 250. A docking grid size of 60 Å × 60 Å × 60 Å with a spacing value of 0.375 Å was employed and centered at the active site of the studied proteins. For the investigated metabolites, SZYBKI software was used to minimize the geometrical structures of the investigated metabolites with the MMFF94s force field [55,56]. The Gasteiger method was utilized to calculate the partial atomic charges of the investigated metabolites [57]. Furthermore, to explore all potential target–function interrelations based on biological network mining for the 20 most stimulated genes by compounds 10 and 29, pathway enrichment analysis (PEA) was performed using Cytoscape 3.8.2 [58]; finally, the ReactomeFIViz online tool was used for the modeling and illustration of all target interactions [59,60].

### 4.7. Statistical Analysis

Statistical analysis was carried out using IBM SPSS^®^ Statistical version 23 (IBM^®^ Corp., Armonk, NY, USA). Data were expressed as mean and standard deviation, and comparison between different groups was performed using analysis of variance (ANOVA) followed by post-hoc test. Dunnett t-test was used for comparison with the control group. All tests were two-tailed. A *p*-value < 0.05 was considered significant.

## 5. Conclusions

*L. sativum* is a promising medicinal plant that requires extensive research in order to reveal its beneficial therapeutic uses and explore its underlying mechanism(s) of action. The results of the current study reveal that the *n*-hexane extract of *L. sativum* showed clear in vitro cytotoxic efficiency against two hepatocellular carcinoma cell lines. This cytotoxic efficiency was further investigated by identifying the expression levels of some apoptotic genes; we observed the significant downregulation of the expression of the EGFR and BCL2 genes and upregulation of the SMAD3, BAX, and P53 genes. This might explain its mechanism of action. Additionally, *n*-hexane extract fractionation demonstrated that hydrocarbons, terpenoids, and volatile compounds were the most predominant. Finally, our molecular docking results against EGFR and BCL2 molecular targets support the potency of *L. sativum*’s major compounds against hepatocellular carcinoma. Ultimately, our findings highlight the potential use of *L. sativum* against HCC.

## Figures and Tables

**Table 1:** Gas chromatography (GC) of *n*-hexane extract.

No.	RT (min)	Compound Name	Formula	M.W	Area%
**Mono-Terpenoids**					**13.38%**
1	11.29	Estragole	C10H12O	148	1.44%
2	13.03	Alpha-terpinyl acetate	C12H20O2	196	2.36%
3	13.27	cis-p-Propenylanisole	C10H12O	148	4.13%
4	18.51	gamma-Asarone	C12H16O3	208	0.68%
5	15.77	Methyleugenol	C11H14O2	178	4.77%
**Sesqui-Terpenoids**					**5.42%**
6	12.3	Copaene	C15H24	204	1.09%
7	13.54	Caryophyllene	C15H24	204	3.82%
8	15.59	Sesquisabinene isomer	C15H24	204	0.74%
9	17.56	Caryophyllene oxide	C15H24O	220	0.86%
**Hydrocarbons**					**69.16%**
10	4.06	Thieno[3,4-c]pyridine-1,3-dione	C31H21NS	439	1.65%
11	19.6	2,4,6-Trimethylmandelic acid	C11H14O3	194	1.41%
12	21.51	Palmitic acid, methyl ester	C17H34O2	270	3.30%
13	23.98	2-Nonadecanone	C19H38O	282	2.40%
14	24.14	Oleic acid, methyl ester	C19H36O2	296	11.76%
15	24.72	Linolenic acid, methyl ester	C19H32O2	292	26.44%
16	26.59	11-Eicosenoic acid, methyl ester	C21H40O2	324	4.80%
17	27.61	Cis-8,11,14-Eicosatrienoic Acid	C20H34O2	306	1.48%
18	27.72	6,9,12,15-Docosatetraenoic acid, methyl ester	C23H38O2	498	4.15%
19	28.88	Methyl (Z)-13-docosenoate	C23H44O2	352	1.18%
20	29.23	2-Hydroxy-1-(hydroxymethyl)ethyl (9z)-9-octadecenoate	C21H40O4	356	1.30%
21	29.84	2-[(9Z,12Z)-9,12-Octadecadien-1-yloxy]ethanol	C20H38O2	310	0.52%
22	31.18	Meadowlactone	C20H38O2	310	2.65%
23	31.46	Dotriacontane	C32H66	450	0.94%
24	33.35	(Z)-18-Octadec-9-enolide	C18H32O2	280	5.18%
**Others**					9.71%
25	14.98	[2-methoxy-4-[(Z)-prop-1-enyl]phenyl] acetate	C10H12O2	164	1.15%
26	18.91	N-[(3-fluorophenyl)methyl]-7H-purin-6-amine	C12H10FN5	243	1.79%
27	30.72	10-Nethoxy-Nb-alpha-methylcorynantheol	C21H29N2O2	341	1.03%
28	28.94	Propanoic acid,2-(3-acetoxy-4,4,14-trimethylandrost-8-en-17-yl)	C27H42O4	430	1.19%
29	35.91	gamma -Tocopherol	C28H48O2	416	3.55%
30	36.79	9,12,15-Octadecatrienoic acid,2-phenyl-1,3-dioxan-5-yl ester	C28H40O4	440	1.00%
**Total**					**97.67%**

**Table 2:** Predicted docking scores (in kcal/mol) for *n*-hexane extract major metabolites with active EGFR, inactive EGFR, and BCL2.

No.	Compound Name	Docking Score (kcal/mol)		
		EGFR		BCL2
		Active	Inactive	
1	Estragole	−4.6	−5.6	−4.2
2	Alpha-Terpinyl acetate	−5.8	−7.7	−5.3
3	Anisole, p-propenyl-, cis-	−4.8	−5.8	−4.4
4	Gamma-Asarone	−4.4	−6.3	−4.2
5	Methyleugenol	−4.5	−6.1	−4.3
6	Copaene	−6.8	−9.0	−6.5
7	Caryophyllene	−6.3	−7.1	−6.0
8	Sesquisabinene isomer	−6.4	−8.4	−6.0
9	Caryophyllene oxide	−6.0	−7.4	−6.1
10	Thieno[3,4-c]pyridine-1,3-dione	−8.5	−12.3	−7.4
11	2,4,6-Trimethylmandelic acid	−5.6	−6.7	−4.5
12	Palmitic acid, methyl ester	−4.7	−8.2	−4.0
13	2-Nonadecanone	−4.8	−9.1	−3.9
14	Oleic acid, methyl ester	−5.3	−8.5	−4.7
15	Linolenic acid, methyl ester	−5.9	−8.7	−4.5
16	11-Eicosenoic acid, methyl ester	−5.4	−7.8	−4.7
17	cis-8,11,14-Eicosatrienoic Acid	−5.3	−9.8	−5.4
18	6,9,12,15-Docosatetraenoic acid, methyl ester	−6.0	−10.3	−4.9
19	Methyl (Z)-13-docosenoate	−3.1	−9.9	−4.5
20	2-Hydroxy-1-(hydroxymethyl)ethyl (9z)-9-octadecenoate	−2.4	−8.3	−4.4
21	2-[(9Z,12Z)-9,12-Octadecadien-1-yloxy]ethanol	−4.6	−8.8	−3.8
22	Meadowlactone	−6.0	−10.1	−5.5
23	Dotriacontane	−4.8	−9.7	−3.4
24	(Z)-18-Octadec-9-enolide	−7.2	−7.9	−7.2
25	2-Methoxy-4-[(1e)-1-propenyl]phenol	−4.7	−6.4	−4.1
26	N-[(3-fluorophenyl)methyl]-7H-purin-6-amine	−6.3	−8.0	−4.9
27	10-Nethoxy-Nb-alpha-methylcorynantheol	−7.9	−7.3	−5.9
28	Propanoic acid,2-(3-acetoxy-4,4,14-trimethylandrost-8-en-17-yl)	−8.0	−5.2	−7.7
29	gamma-Tocopherol	−8.5	−12.3	−7.0
30	9,12,15-Octadecatrienoic acid, 2-phenyl-1,3-dioxan-5-yl ester	−6.3	−11.7	−6.8

## Data Availability

All data, tables and figures in this manuscript are original. If not, please provide the copyright.

## Supplementary Materials

The following are available online at https://www.mdpi.com/article/10.3390/plants10091863/s1, Figure S1: A Reactome map of the top enriched pathways affected by the top 20 gene targets in response to compound 10. The yel-low color code indicates the over-representation of that pathway in the input dataset. Light grey signifies pathways that are not significantly over-represented. Figure S2: A Reactome map of the top enriched pathways affected by the top 20 gene targets in response to compound 29. The yel-low color code indicates the over-representation of that pathway in the input dataset. Light grey signifies pathways that are not significantly over-represented.
