# Supplementary material for: Lepidium sativum Secondary Metabolites (Essential Oils): In Vitro and In Silico Studies on Human Hepatocellular Carcinoma Cell Lines
Source: Plants (Basel). 2021 Sep 9;10(9):1863. doi: 10.3390/plants10091863 (PMC8470406; doi:10.3390/plants10091863)
Supplement: Supplementary file 1 [file plants-10-01863-s001.zip › plants-1362056-supplementary.pdf]

plants

Supplementary Materials

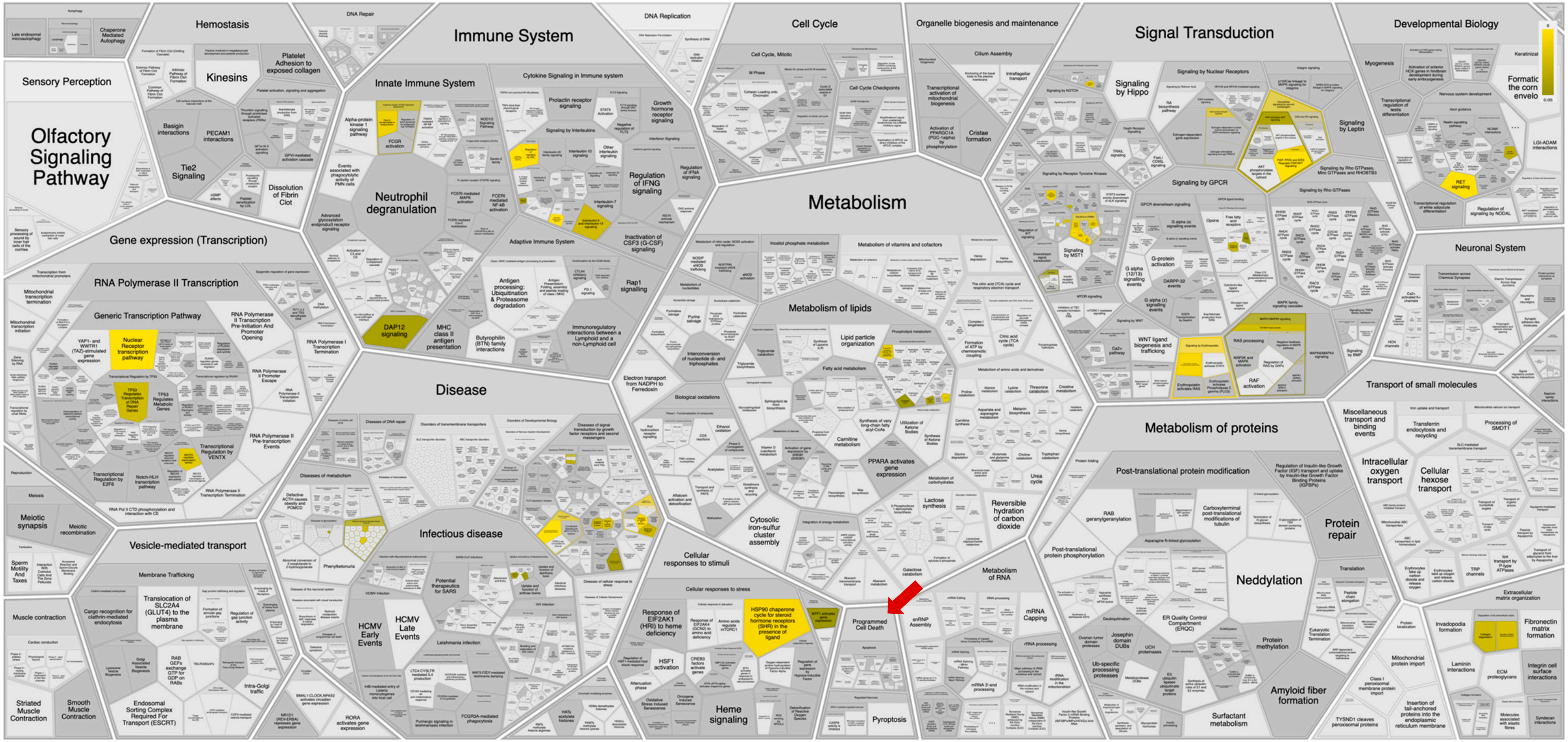

Figure S1. A Reactome map of the top enriched pathways affected by the top 20 gene targets in response to compound 10 . The yellow color code indicates the over-representation of that pathway in the input dataset. Light grey signifies pathways that are not significantly over-represented.

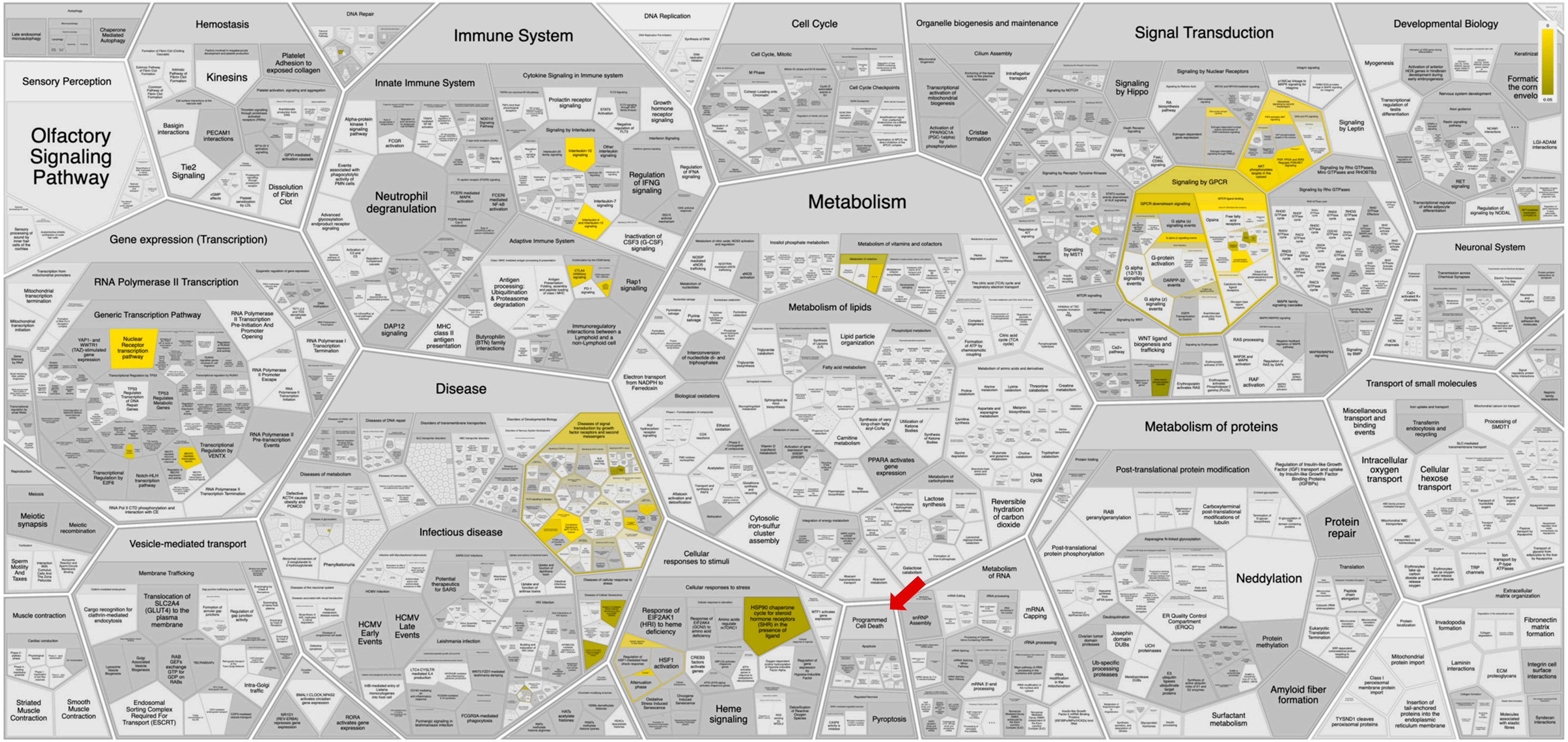

Figure S2. A Reactome map of the top enriched pathways affected by the top 20 gene targets in response to compound 29. The yellow color code indicates the over-representation of that pathway in the input dataset. Light grey signifies pathways that are not significantly over-represented.
